# Utilization of Information Communication Technology and Its Associated Factors Among Healthcare Professionals: Systematic Review and Meta‐Analysis, in the Resource‐Limited Setting

**DOI:** 10.1155/bmri/1329276

**Published:** 2026-02-24

**Authors:** Adamu Ambachew Shibabaw, Fikadu Wake Butta, Alex Ayenew Chereka, Yosef Haile Gebreariam, Gemeda Wakgari Kitil, Daniel Niguse Mamo

**Affiliations:** ^1^ Department of Health Informatics, College of Medicine and Health Science, Debre Berhan University, Debre Berhan, Ethiopia, dbu.edu.et; ^2^ Department of Health Informatics, College of Health Science, Mattu University, Mattu, Ethiopia, meu.edu.et; ^3^ Department of public Health, College of Medicine and Health Sciences, Arba Minch University, Arba Minch, Ethiopia, amu.edu.et; ^4^ Department of Midwifery, College of Health Science, Mattu University, Mattu, Ethiopia, meu.edu.et; ^5^ Department of Health Informatics, College of Medicine and Health Science, Arba Minch University, Arba Minch, Ethiopia, amu.edu.et

**Keywords:** communication, healthcare professional, information, technologies, utilization

## Abstract

**Introduction:**

The term “information and communication technologies” encompasses a broad array of digital and electronic devices that facilitate the sharing, processing, transmission, and communication of health information and knowledge.

**Method:**

A search of the literature was conducted using databases such as Google Scholar, HINARI, PubMed, Scopus, EMBASE, Web of Science, African Journal Online, and Global Health. Search engines were used to locate studies that adhered to the PRISMA Protocols. STATA Version 11 was used for analysis, with heterogeneity assessed using the Cochrane Qtest, *p* values, and *I* ^2^ statistics. The meta‐analysis showed no significant heterogeneity for pooled information and communication technologies utilization prevalence, prompting the use of a fixed‐effect model. Heterogeneity was assessed using forest plots, and funnel plot asymmetry and the Egger regression test were used to evaluate publication bias.

**Results:**

Out of 18,924 publications reviewed, 10 studies with 4,171 health workers met the inclusion criteria for the systematic review and meta‐analysis. Informationprevalence was found to be 41.92% (95% CI: 33.47, 50.37). Knowledge of ICT 4.47 times (AOR = 4.47, 95% CI: 2.69, 7.42), ICT training 3.43 times (AOR = 3.43, 95% CI: 2.11, 5.57), educational status 2.84 times (AOR = 2.84, 95% CI: 1.80, 4.47), basic computer skills 2.99 times (AOR = 2.99, 95% CI: 2.89, 17.11), attitudes 7.07 times (AOR = 7.07, 95% CI: 2.89, 17.11), computer access 7.15 times (AOR = 7.15, 95% CI: 4.66, 10.97), and urban residents 10 times (AOR = 10.00, 0.29, 347.80) were found to be associated with information and communication technology utilization.

**Conclusion:**

The utilization of Ethiopia is low, highlighting the necessity for government investment in training for the effective use of these technologies.

## 1. Introduction

Information and communication technology (ICT) is a comprehensive array of electronic tools and technologies—such as radio, television, telephones (both fixed and mobile), computers, the Internet, and wireless networks—that facilitate the processing, storage, transmission, and communication of information. In the field of health, ICT is utilized to enhance health services, health systems, surveillance, education, and research [1, 2]. Advancements in ICT have sparked a revolution in various domains, including communication, education, healthcare, and business practices [3]. The widespread availability and easy accessibility of ICT tools like smartphones, computers, and the Internet have fundamentally transformed how individuals interact and access information [4, 5].

With an estimated investment of over 1 trillion dollars to date on information technology (IT) products and applications, one would hope that there exists a corresponding improvement in organizational performance and productivity [1].

ICT has firmly established itself as an indispensable component of modern society, with far‐reaching implications for individuals, communities, and societies at large [6]. It is increasingly prevalent in education, healthcare, and business, driven by advancements in communication systems that have significantly influenced our daily lives, including the healthcare sector, and is also directly related to education. When used effectively, it can assist both the general education and training processes and the students in the classroom [7]. Moreover, it has emerged as a potent instrument for addressing global poverty, offering developing nations an unparalleled chance to achieve crucial development objectives, including poverty alleviation, basic healthcare, and education, with greater efficacy than ever before [5, 8–10].

As ICT use in healthcare continues to expand, it enhances patient independence and decreases hospital stays, which in turn reduces the overall cost of care since earlier discharge also allows more patients to be treated with the same number of beds; therefore, understanding the factors that influence the adoption and effective use of these technologies has become increasingly important [1]. Additionally, numerous countries are leveraging ICT to modernize their healthcare sector, utilizing it for various purposes such as telemedicine, health sector management, health information databases, and health education [11]. The potential of ICTs to significantly enhance access and quality of healthcare services is substantial [12]. Recognizing this role, researchers have acknowledged ICT′s capacity to facilitate growth and development [13]. Understanding the pooled factors influencing ICT utilization is essential for policymakers, researchers, and practitioners in various domains [14]. Identifying these factors provides valuable insights into the determinants of ICT adoption and usage patterns, informing the development of effective strategies to bridge the digital divide and promote equitable access to technology.

ICT is playing a transformative role in patient care, particularly through innovative telemedicine projects that enable remote assistance by regional healthcare systems. These technologies support patients in their homes, enhance rehabilitation processes, and help monitor motor complications while strengthening both clinical skills and knowledge. ICT‐based strategies are increasingly used for effective patient management and for providing targeted education to help individuals cope with daily challenges and disease‐related difficulties. Despite these advances, several studies consistently highlight that caregivers continue to face significant psychophysical overload, characterized by high levels of anxiety, perceived stress, loneliness, reduced social engagement, and an overall decline in quality of life [2].

The findings of this systematic review and meta‐analysis have implications for various stakeholders involved in promoting digital inclusion, technology adoption, and ICT utilization. Policymakers can develop evidence‐based policies and initiatives that address barriers and promote equitable access by comprehensively understanding the factors influencing ICT utilization. This systematic review and meta‐analysis can identify pooled results and help to investigate the gap, whereas practitioners can develop targeted interventions and strategies to enhance ICT utilization in specific contexts or populations. This review is specifically aimed at analyzing and synthesizing the existing literature on the utilization of ICT in healthcare, identifying the factors influencing its adoption and implementation, and assessing the impact of ICT on healthcare outcomes and patient care.

## 2. Methods

### 2.1. Search Strategy

Between August 1, 2024, and December 12, 2024, a comprehensive and methodical search of the literature was conducted using electronic databases such as PubMed, HINARI, Global Health, Scopus, EMBASE, Web of Science, African Journal Online (AJOL), and Google Scholar. The keywords “utilization” or “practice” and “information communication technology” or “ICT” and “associated factors” or “determinants” and “healthcare professionals” or “healthcare workers” or “healthcare facilities” and “Ethiopia” were used in the search. Extraction of data was carried out using a standardized data extraction technique that was modified from the Joanna Briggs Institute (JBI) following a screening of the titles, abstracts, and full texts of each included original study. Following the independent extraction of data, each included publication was scrutinized by two reviewers (A.D.W. and A.A.S.). The initial author′s name, the study′s location and setting, the year it was published, the study′s design, the participants, the sampling strategy, the data source, the sample size, and the response rate were among the attributes of the study that were retrieved. ICT prevalence (utilization) and risk factors were also extracted along with their 95% confidence intervals.

### 2.2. Eligibility Criteria

Original research studies that discussed the utilization of ICT among Ethiopian health workers and associated factors were included in the study. Cross‐sectional studies were taken into consideration, regardless of the year of publication. Articles published up until December 12, 2024 were considered. Moreover, all cross‐sectional research with a primary focus on ICT utilization in the Ethiopian healthcare system are included in this systematic review, and this meta‐analysis excluded articles lacking full text and abstracts. This review contains readily accessible full‐text English publications that can be discovered in the grey literature or published in peer‐reviewed journals. Studies published in languages other than English or studies that are not cross‐sectional are not included in this systematic review.

### 2.3. Outcome Measurement

This systematic review and meta‐analysis′ outcome was an the evaluation of Ethiopia′s healthcare professionals′ ICT utilization. These variables were quantified using odds ratios (AOR). The binary outcome data provided by each primary study were used to compute the odds ratio for each component that was found.

### 2.4. Evaluation of the Selected Literature′s Quality

The original studies′ quality was assessed using the Newcastle–Ottawa scale, a method created for cross‐sectional study quality evaluations. Three primary components make up the assessment maximum of three‐star selection: (maximum of 3∗).

1. Representative of the sample

• Truly representative of the average population in the target (all subjective or random sampling).
• Somewhat representative of the average population in the target (nonrandom sampling).

2. Selected group of users

• No description of the sampling strategy.

3. Nonrespondents

• Comparability between nonrespondents and respondents′ characteristics is established, and the response rate is satisfactory.
• The response rate is unsatisfactory, or the comparability between nonrespondents and respondents is unsatisfactory.
• No description of the response rate, of the characteristics of nonrespondents, and respondents′.

#### 2.4.1. Assessment of Exposure (Risk Factors)

1. Valid assessment tools

• Nonvalid assessment tools, and the tools that are available or described.

2. No description of the assessment tools

#### 2.4.2. Comparability (Maximum 2∗)

The subjects in the different outcome groups are comparable based on the study design or analysis.

4. Confounding factors are controlled

• The study controls the most important factor (select)
• The study controls for any additional factors

#### 2.4.3. Outcome (Maximum 2∗)

Assessment of outcome:

• independent blind assessment
• record linkage
• self‐report
• no description

#### 2.4.4. Statistical Test

1. The statistical test used to analyze the data is clearly described and appropriate, and the measurement of the association is presented, including confidence interval and the probability level (*p* value).
2. The statistical test is not appropriate to describe or is incomplete. The review′s two writers, A.A.C and G.W.K., evaluated the research′s quality on their own. Discussions were used to resolve disagreements between reviewers during the quality evaluation process (Table S1).

Key: Y, yes; NR, not reported, NA, not appropriate

Question codes:

1. Was the sample frame appropriate to address the target population?
2. Were study participants sampled in an appropriate way?
3. Was the sample size adequate?
4. Were the study subjects and the setting described in detail?
5. Was the data analysis conducted with sufficient coverage of the identified sample?
6. Were valid methods used for the identification of the condition?
7. Was the condition measured in a standard, reliable way for all participants?
8. Was there an appropriate statistical analysis?
9. Was the response rate adequate, and if not, was the low response rate managed appropriately?

### 2.5. Data Extraction

Three investigators constructed a common computer‐based spreadsheet with the data from the included studies, whereas another researcher verified its correctness. For every study, information was gathered on the initial author, the year the study was published, the number of participants, the study areas and types of participants, the sample size, the data collection techniques, and the study design. The prevalence of ICT utilizations and associated factors was also retrieved and adjusted odds ratio with 95% confidence intervals.

### 2.6. Data Synthesis and Analysis

In the present review, the data analysis was performed using STATA Version 11 Statistical Software. The data were extracted in Microsoft Excel (the logarithm of the odds ratio and the standard error of the logarithm of the odds ratio of each study were calculated and exported in STATA for further analysis. The heterogeneity across studies was assessed by using the Cochrane *Q* test statistics, *p* value and *I* ^2^ (*I*‐squared) test statistics. It was considered as low, moderate, or high when *I* ^2^ test statistics result was 25%, 50%, and 75%, respectively. Visually, we used a forest plot to assess the presence of heterogeneity. In all cases, a *p* value of less than or equal to 0.05 was considered to be statistically significant heterogeneity. The finding of the meta‐analysis was presented using the forest plot and odds ratio with its 95% CI. Visual inspection of symmetry in funnel plots and Egger regression tests was employed to assess the existence of publication bias.

## 3. Result

### 3.1. Search Results

**Figure 1:**
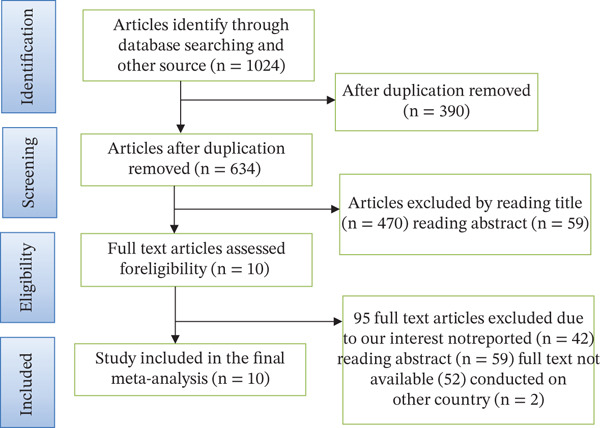
Flow chart of study selection for systematic review and meta‐analysis utilization of information communication (ICT) and associated factors among health workers in Ethiopia.

A total of 1924 articles regarding the utilization of information communication and associated factors among health workers in Ethiopia were retrieved (Figure 1).

### 3.2. Characteristics of the Eligible Studies

Each of the included studies estimated ICT utilization of a facility‐based cross‐sectional survey approach. Every study that was a part of this review was released no later than December 12, 2024. Of the 10 studies that made up this review, two employed multistage sampling, one utilized cluster sampling, one used purposive sampling, and one used systematic random sampling. Nine of the included studies employed a self‐administered method for participant selection, and one study utilized an interviewer‐administered method. A total of 4171 persons were included, and the expected range of sample sizes (Table 1).

**Table 1:** Descriptive summary of primary studies included in the meta‐analysis of the utilization of information communication technology and associated factors among healthcare workers.

First author, publication year	Region	Study area	Study design	Study population	Sampling technique	Data collection method	Sample size	R‐rate
Belay [15] 2017.	Amhara	Specialized Hospital	FBCS	HP	Simple random	Structured Self‐administered	314	94.9%
Hailegebreal et al. [16] 2022	SNNP	Arba Minch University	FBCS	Health sciences students	Simple random	Self‐administered	355	98.3%
Woreta et al. [8] 2013	Amhara	Specialized Hospital	FBCS	Health sciences students	Cluster sampling	Self‐administered	1096	97.8%
Mohammed et al. [17] 2013	A A	Addis Ababa	IBCS	HP	Stratified	Self‐administered	270	88.8%
Demsash et al. [18] 2023	Oromia	Mettu University	IBCS	Health science students	Stratified simple random	Self‐administrated	412	97.4%
Tsigie et al. [19] 2021	Amhara	Debretabour Referral Hospital	IBCS	HP	Simple random	Self‐administered	314	94.9%
Tsigie et al. [20] 2019	Amhara	Bahirdar University	IBCS	Health sciences Academic Staffs	Simple random	Self‐administered	351	100%
Alwan et al. [21] 2015	Harar	Harar Health Science College	IBCS	HP	Simple random	Structured, self‐administered	482	87.0%
Buta [22] 2016	Oromia	Haramaya university	Mixed	HP	Stratified proportional random, purposively	Structured In‐depth interview key informants	265	94.3%
Asemahagn [23] 2016	Addis Ababa	Hospitals in Addis Ababa	IBCS	HP	Selected systematically	Self‐administered	312	97.5%

### 3.3. Utilization of ICT Among Health Workers in Ethiopia

Overall, the pooled estimate of the utilization of ICT among health workers in Ethiopia was 41.92% (95% CI: 33.47, 50.37). This meta‐analysis displayed that there is no statistically significant heterogeneity among studies (*I* ^2^ = 18.6, *p* = 0.258); therefore, the fixed effect model was applied to estimate the pooled estimate of ICT utilization.

### 3.4. Factors Associated With Utilization Information and Communication

**Figure 2:**
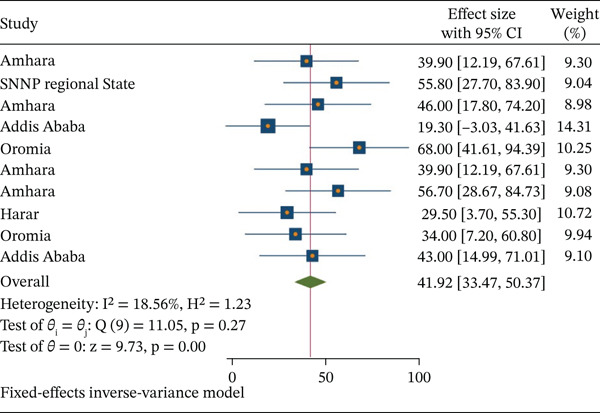
Forest plot of pooled prevalence of information communication technology (ICT) among health workers in Ethiopia.

Three studies found a significant connection between the age groups of (20–25) [19, 24] and (26–35) [25] and the use of ICT. Health care personnel who received training were 2.98 times (AOR = 2.98; 95*%*CI : 1.65, 5.39) more likely to use ICT. Knowledge of ICT is 4.47 times (AOR = 4.47; 95*%*CI : 2.69, 7.42) more common among health personnel than those with poor ICT knowledge. Four studies additionally demonstrated the correlation between ICT use and ICT training [19, 24–27]. Compared with their counterparts, health workers with ICT training had odds of using ICT that were 3.43 times ( AOR = 3.43; 95*%*CI : 2.11 , 5.57) higher. Five studies have shown that the use of ICT has a correlation with educational status [19, 24, 25, 27, 28]. Compared with their counterparts, health workers with ICT training had odds of using ICT that were 3.43 times ( AOR = 3.43 ; 95*%*CI : 2.11 , 5.57) higher. Five studies have shown that the use of ICT has a correlation with educational status [8, 15, 19, 24, 25, 27, 28], degree, and above. ICT use was associated with educational status and was 2.84 times (AOR = 2.84; 95*%*CI : 1.80, 4.47) higher for health workers with their counterparts. Three studies showed that the use of ICT is associated with basic computer skills [15, 17, 25]. When comparing health workers with basic computer skills to their counterparts, the odds of using ICT were around 2.99 times (AOR = 2.99; 95*%*CI : 2.20, 4.06) higher. Two studies showed the association between attitude and the use of ICT [26, 29]. When comparing health workers with good attitudes, the odds of using ICT were around 7.07 times (AOR = 7.07; 95*%*CI : 2.89, 17.11) higher than their counterparts. When comparing health workers with different attitudes, the odds of using ICT were around 7.07 times (AOR = 7.07; 95*%*CI : 2.89, 17.11) higher. Two studies demonstrated the relationship between computer access and the use of ICT [26, 29]. Compared with their counterparts, health workers with computer access had probabilities of using ICT that were 7.15 times (AOR = 7.15; 95*%*CI : 4.66, 10.97) higher. Furthermore, two studies [16, 22] show that the use of ICT is associated with the previous resident. Therefore, compared with their counterparts, healthcare providers who lived in cities had a higher likelihood of using ICT. The utilization of ICT among health workers in Ethiopia is presented in Figure 2.

### 3.5. Publication Bias

**Figure 3:**
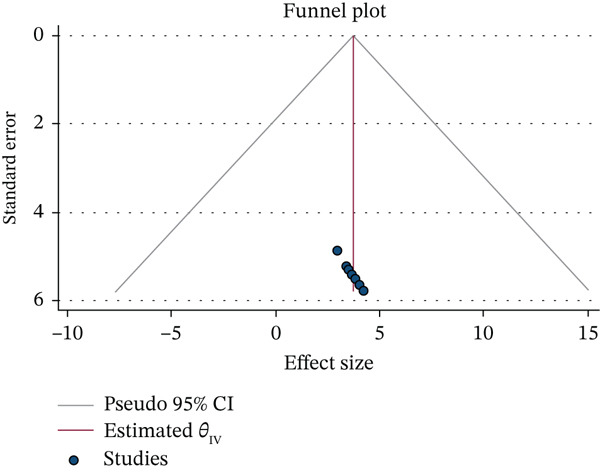
Graphical representation of publication bias using a funnel plot of information communication technology (ICT) among health workers in Ethiopia.

To determine whether publication bias existed, Egger regression tests and visual examination of the asymmetry in funnel plots were used. As a result, the results of Egger′s tests and funnel plots demonstrated that the included papers did not exhibit publication bias. The absence of publication bias was stated by Egger′s test result, which was not statistically significant (*p* = 0.193). Furthermore, a symmetric distribution of studies was visible upon visual inspection of the funnel plots (Figure 3).

## 4. Discussion

The study of pooled prevalence showed that 41.92% (95*%*CI : 33.47, 50.37) of Ethiopian health professionals utilized ICT. This pooled prevalence was aligned with confidence interval research conducted at Gondar Comprehensive Specialized Hospital (39.9%), Addis Ababa (19.3%), Harare (29.5%) [30], and a Côte d’Ivoire study that discovered 38% of adults utilized ICT [3]. Nonetheless, this study′s outcome was lower than those of studies carried out in Texas (81%), Austria (75%), the United States (91%), and Belgium, where 94% of working healthcare professionals are in psychiatric hospitals and 93% of general acute care hospitals have good ICT usage habits. These differences might be attributed to the infrastructure and economic status of the countries, with other studies carried out in Tanzania (58%) [20] and South Africa (65%) [21]. This difference may be a result of variations in the structures of health information systems and the attitudes of healthcare professionals toward the use of ICT. This study indicated that the age group 17, 24–27, 29 [1, 6] and the age group 26–35 [8] have a significant association with the utilization of ICT and were more likely to use ICT as compared with those with other lower age groups. This finding was supported by other studies conducted in Tanzania [2] and rural South Africa [30].

Additionally, this study showed that health personnel who knew about ICT were 4.47 times more likely to use them than those who did not. This may be because professionals are aware of the benefits of ICT for their everyday tasks. The odds of utilization of ICT were about 3.43 times higher among health workers who have ICT training than their counterparts. The result was consistent with previous studies in Ethiopia and India [19, 28]. This could be because the presence of ICT training can help health workers utilize ICT for evidence‐based decision‐making.

**Figure 4:**
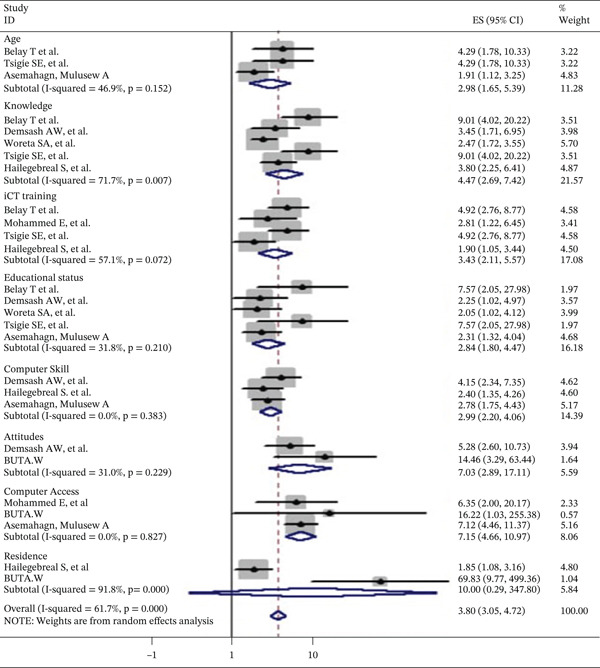
Associated factors of studies included in the meta‐analysis of information communication technology (ICT) among health workers in Ethiopia.

Health workers with a degree or higher education were 2.84 times more likely to utilize ICT compared with those with lower educational qualifications. This finding is supported by other previous studies done in Medical College, Burla, India [17]. This may be because having a degree or higher equips health workers with essential skills to process and manage health information using ICT, highlighting that education is critical for effective ICT utilization. The odds of utilization of ICT were about 3.45 times higher among trained health workers when compared with their counterparts. This finding is supported by other studies [22]. This may be because health workers who receive basic ICT training gain a clearer understanding of its importance, which helps them more effectively use ICT for collecting, compiling, analyzing, and applying information in their daily activities. Health workers who had basic computer skills were 2.99 times more likely to utilize ICT than those who were not skilled. The finding was supported by a similar study [26]. This could be attributed to the fact that knowing how to use a range of computer programs, software, and other applications is an important IT ability. Like Word processing, spreadsheets, databases, PowerPoint presentations, and search engines are just a few examples of ICT applications that having basic computer skills may aid with. This finding was supported by a study from a previous study [24]. Having basic ICT skills is important for effective ICT use, as workers with such training better understand its value and are more capable of using computers and electronic tools in their daily activities. The variable “health worker′s attitude,” which is identified as a factor affecting ICT utilization in this study, confirms the study result [27]. Regarding HW residence, the study finding is similar to that of [25], which identified variables that influence internet adoption, such as geographical factors like residency in an urban area and rural community. Similarly, participants who had their own computers were more likely to have adequate utilization of ICT than those who did not own computers [9]. This is also true or consistently in line with the saying that “innovation can be adopted after knowing the innovation to be adopted.” When compared to healthcare professionals from rural areas, healthcare professionals from previous residences in urban areas were more likely to utilize ICT. This finding was similar to a previous study [29]. This might be due to the fact that healthcare professionals from rural residences generally exhibit less ICT usage than urban health workers, mainly because rural professionals often lack access to essential infrastructure such as electricity, computers, and other electronic resources (Figure 4).

## 5. Limitations of the Study

Despite being the first systematic review and meta‐analysis on the topic, this study has some drawbacks. First, only English‐language publications were taken into account in this meta‐analysis. Second, with cross‐sectional studies that were present in every manuscript under consideration were facility‐based, which limited how broadly the findings could be applied. Moreover, the present analysis just encompassed research from five distinct locations, potentially impacting the aggregate frequency of ICT utilization.

## 6. Conclusion

According to the survey, the majority of healthcare workers used ICT infrequently; however, those who had a positive attitude toward ICT demonstrated high levels of utilization. Knowledge of information and communication technologies were also relevant. Better use of ICT applications was more likely among those with higher educational degrees.

## 7. Recommendation

The Ethiopian government should strengthen ICT infrastructure, especially in rural health facilities, by expanding reliable electricity, internet access, and computer availability. In addition, continuous ICT training programs should be provided for health workers at all levels to build essential digital skills. Prioritizing policy support, budget allocation, and partnerships with ICT stakeholders will help create an enabling environment that increases ICT adoption and improves the overall efficiency and quality of healthcare services. This will provide opportunities for healthcare professionals to pursue higher education, as professionals develop healthy ICT habits.

**Nomenclature**
AA: Addis Ababa
AOR: adjusted odd ratio
CI: confidence interval
ICT: information communication technology
HP: healthcare professional
IBCS: institutional based cross‐sectional
FBCS: facility‐based cross‐sectional
PRISMA: preferred reporting items for systematic reviews and meta‐analyses
NOS: Newcastle–Ottawa scale
SNNP: South Nation Nationality People

## Author Contributions

A.A.S., D.N.M., and Y.H.G. were responsible for study selection, conceptualization, data extraction, statistical analysis, and the initial drafts of the publication. A.A.S., A.A.C., F.W.B., D.N.M., and G.W.K. GWK wrote the final draft of the manuscript.

## Funding

No funding was received for this manuscript.

## Disclosure

All authors read and approved the final version.

## Ethics Statement

The authors have nothing to report.

## Conflicts of Interest

The authors declare no conflicts of interest.

## Supporting Information

Additional supporting information can be found online in the Supporting Information section.

## Supporting information


**Supporting Information 1** Preferred Reporting Items for Systematic Reviews and Meta‐Analyses (PRISMA) checklist.

**Supporting Information 2** Table S1: Quality assessment of utilization of information communication technology and its associated factors among healthcare professionals systematic review and meta‐analysis in the resource‐limited setting.

## Data Availability

The corresponding author can provide any data generated or analyzed during this study upon reasonable request.

## References

[1] Crowther M. , Lim W. , and Crowther M. A. , Systematic Review and Meta-Analysis Methodology, Blood. (2010) 116, no. 17, 3140–3146, 10.1182/blood-2010-05-280883, 2-s2.0-78049398953, 20656933. 20656933

[2] Alpay L. and Russell A. , Information Technology Training in Primary Care the Nurses’ Voice, CIN: Computers, Informatics, Nursing. (2002) 20, no. 4, 136–142. 10.1097/00024665-200207000-00008 12105401

[3] Darwish A. and Lakhtaria K. I. , The Impact of the New Web 2.0 Technologies on Communication, Development, and the Revolutions of Societies, Journal of Advances in Information Technology. (2011) 2, no. 4, 204–216.

[4] Shao M. , Fan J. , Huang Z. , and Chen M. , The Impact of Information and Communication Technologies (ICTs) on Health Outcomes: A Mediating Effect Analysis Based on Cross-National Panel Data, Journal of Environmental and Public Health. (2022) 2022, 2225723, 10.1155/2022/2225723. 35990542 PMC9385304

[5] Joshi A. , Meza J. , Costa S. , Perin D. M. P. , Trout K. , and Rayamajih A. , The Role of Information and Communication Technology in Community Outreach, Academic and Research Collaboration, and Education and Support Services (IT-CARES), Perspectives in Health Information Management. (2013) 10, 24159275. PMC3797554 24159275

[6] Thompson J. B. , The Media and Modernity a Social Theory of the Media, 1995, Stanford University Press.

[7] Dwivedi Y. K. , Hughes L. , Ismagilova E. , Aarts G. , Coombs C. , Crick T. , Duan Y. , Dwivedi R. , Edwards J. , Eirug A. , Galanos V. , Ilavarasan P. V. , Janssen M. , Jones P. , Kar A. K. , Kizgin H. , Kronemann B. , Lal B. , Lucini B. , Medaglia R. , le Meunier-FitzHugh K. , le Meunier-FitzHugh L. C. , Misra S. , Mogaji E. , Sharma S. K. , Singh J. B. , Raghavan V. , Raman R. , Rana N. P. , Samothrakis S. , Spencer J. , Tamilmani K. , Tubadji A. , Walton P. , and Williams M. D. , Artificial Intelligence (AI): Multidisciplinary Perspectives on Emerging Challenges, Opportunities, and Agenda for Research, Practice and Policy, International Journal of Information Management. (2021) 57, 101994, 10.1016/j.ijinfomgt.2019.08.002, 2-s2.0-85071255877.

[8] Woreta S. A. , Kebede Y. , and Zegeye D. T. , Knowledge and Utilization of Information and Communication Technology (ICT) Among Health Science Students at the University of Gondar, North Western Ethiopia, BMC Medical Informatics and Decision Making. (2013) 13, no. 1, 1–7. 23452346 10.1186/1472-6947-13-31 PMC3599196

[9] Mikre F. , The Roles of Information Communication Technologies in Education: Review Article With Emphasis to the Computer and Internet, Ethiopian Journal of Education and Sciences. (2011) 6, no. 2, 109–126, 10.4314/ejesc.v6i2.

[10] Dharma W. R. , Copriady J. , and Linda R. , The Utilization of ICT as Pedagogical and Professional Competencies to Support the Professionalism of Chemistry Teachers, Indonesian Research Journal in Education| IRJE. (2020) 4, 291–305, 10.22437/irje.v4i2.9107.

[11] While A. and Dewsbury G. , Nursing and Information and Communication Technology (ICT) a Discussion of Trends and Future Directions, International Journal of Nursing Studies. (2011) 48, no. 10, 10.1016/j.ijnurstu.2011.02.020, 2-s2.0-80053219273, 21474135. 21474135

[12] Perron B. E. , Taylor H. O. , Glass J. E. , and Margerum-Leys J. , Information and Communication Technologies in Social Work, Advances in Social Work. (2010) 11, no. 2, 67–81, 21691444. 21691444 PMC3117433

[13] Scheffler E. , Poverty in the Book of Proverbs: Looking From Above?: Old Testament Wisdom, Human Dignity and the Poor, Scriptura: Journal for Contextual Hermeneutics in Southern Africa. (2012) 111, no. 1, 480–496.

[14] Almaiah M. A. , Al-Khasawneh A. , and Althunibat A. , Exploring the Critical Challenges and Factors Influencing the E-Learning System Usage During COVID-19 Pandemic, Education and Information Technologies. (2020) 25, no. 6, 5261–5280, 10.1007/s10639-020-10219-y, 32837229. 32837229 PMC7243735

[15] Belay T. , Knowledge, Attitude and Utilization of Information Communication Technologies (ICTs) Among Health Professionals at University of Gondar Comprehensive Specialized Hospital, Northwest Ethiopia, 2017, 10.20372/NADRE:1547201724.48.

[16] Hailegebreal S. , Sedi T. T. , Belete S. , Mengistu K. , Getachew A. , Bedada D. , Molla M. , Shibiru T. , and Mengiste S. A. , Utilization of Information and Communication Technology (ICT) Among Undergraduate Health Science Students: A Cross-Sectional Study, BMC Medical Education. (2022) 22, no. 1, 1–7, 10.1186/s12909-022-03296-9. 35354457 PMC8965211

[17] Mohammed E. , Andargie G. , Meseret S. , and Girma E. , Knowledge and Utilization of Computers Among Health Workers in Addis Ababa Hospitals, Ethiopia Computer Literacy in the Health Sector, BMC Research Notes. (2013) 6. 10.1186/1756-0500-6-106 PMC3610130 23514191

[18] Demsash A. W. , Emanu M. D. , and Walle A. D. , Digital Technology Utilization and its Associated Factors Among Health Science Students at Mettu University, Southwest Ethiopia: A Cross-Sectional Study, Informatics in Medicine Unlocked. (2023) 38, 101218.

[19] Tsigie S. E. and Dagnaw G. A. , Knowledge and Utilization of Information Communication Technologies (ICTs) Among Health Professionals at Debretabor Referral Hospital, Northwest Ethiopia, International Journal. (2021) 10, no. 2, 1324–1330, 10.30534/ijatcse/2021/1191022021.

[20] Tsigie S. E. and Dagnaw G. A. , Utilization of Information Communication Technology and Associated Factors Among Academic Staff in Bahirdar University, North West Ethiopia, 2019, International Journal of Computer Applications. (2019) 975.

[21] Alwan K. , Ayele T. A. , and Tilahun B. , Knowledge and Utilization of Computers Among Health Professionals in a Developing Country: A Cross-Sectional Study, JMIR Human Factors. (2015) 2, no. 1, e4184, 10.2196/humanfactors.4184. PMC4797659 27025996

[22] Buta M. W. , Factors Affecting Access and Utilization of Information Communication Technologies Among Health Workers the Case of West Arsi Zone Government Health Institutions, Oromia, Ethiopia, 2016.

[23] Asemahagn M. A. , Health Professionals’ Challenge in Using ICTs to Manage Their Patients: The Case of Hospitals in Addis Ababa, Ethiopia, Online Journal of Nursing Informatics (OJNI). (2016) 20, no. 2.

[24] Meso P. , Musa P. , and Mbarika V. , Towards a Model of Consumer Use of Mobile Information and Communication Technology in LDCs: The Case of Sub-Saharan Africa, Information Systems Journal. (2005) 15, no. 2, 119–146.

[25] Birba O. and Diagne A. , Determinants of Adoption of the Internet in Africa: Case of 17 Sub-Saharan Countries, Structural Change and Economic Dynamics. (2012) 23, no. 4, 463–472.

[26] Obayelu A. and Ogunlade I. , Analysis of the Uses of Information Communication Technology (ICT) for Gender Empowerment and Sustainable Poverty Alleviation in Nigeria, International Journal of Education and Development Using ICT. (2006) 2, no. 3, 45–69.

[27] Afolayan O. and Oyekunle R. , Availability, Accessibility, and Frequency of Use of ICT Tools by Health Professionals in Ilorin Metropolis, Covenant Journal of Informatics and Communication Technology. (2014) 2, no. 1.

[28] Maharana B. , Biswal S. , and Sahu N. , Use of Information and Communication Technology by Medical Students: A Survey of VSS Medical College, Burla, India, Library Philosophy and Practice (e-journal). (2009) 281.

[29] Gagnon M.-P. , Desmartis M. , Labrecque M. , Car J. , Pagliari C. , Pluye P. , Frémont P. , Gagnon J. , Tremblay N. , and Légaré F. , Systematic Review of Factors Influencing the Adoption of Information and Communication Technologies by Healthcare Professionals, Journal of Medical Systems. (2012) 36, no. 1, 241–277, 10.1007/s10916-010-9473-4, 2-s2.0-84860233032, 20703721. 20703721 PMC4011799

[30] Benard R. , Dulle F. , and Lamtane H. , Challenges Associated With the Use of Information and Communication Technologies in Information Sharing by Fish Farmers in the Southern Highlands of Tanzania, Journal of Information, Communication and Ethics in Society. (2020) 18, no. 1, 44–61.

