# Supplementary material for: Utilization of Information Communication Technology and Its Associated Factors Among Healthcare Professionals: Systematic Review and Meta‐Analysis, in the Resource‐Limited Setting
Source: Biomed Res Int. 2026 Feb 24;2026:1329276. doi: 10.1155/bmri/1329276 (PMC12930299; doi:10.1155/bmri/1329276)
Supplement: Supplementary file 2 — Supporting Information 2 Table S1: Quality assessment of utilization of information communication technology and its associated factors among healthcare professionals systematic review and meta‐analysis in the resource‐limited setting. [file BMRI-2026-1329276-s001.docx]

**S1 Table: Quality assessment** **Utilization of Information Communication Technology and Its Associated Factors among Healthcare Professionals: Systematic Review and Meta-analysis, in the resource-limited setting**

| Author, year of  Study | Q1 | Q2 | Q3 | Q4 | Q5 | Q6 | Q7 | Q8 | Q9 | Total score (9%) |
| --- | --- | --- | --- | --- | --- | --- | --- | --- | --- | --- |
| Belay T, 2017. | Y | Y | Y | Y | Y | Y | NA | Y | Y | 8 |
| Hailegebreal S , et al 2022 | Y | Y | Y | Y | Y | NA | Y | NA | Y | 7 |
| Woreta SA, et al 2013 | Y | Y | Y | Y | Y | Y | Y | Y | Y | 9 |
| Mohammed E, et al 2013 | Y | Y | Y | Y | Y | Y | Y | Y | Y | 9 |
| Demsash AW, et al 2023 | Y | Y | Y | NA | Y | Y | Y | Y | Y | 8 |
| Tsigie SE, et al 2021 | Y | Y | Y | Y | Y | Y | Y | Y | Y | 9 |
| Tsigie SE,et al 2019 | Y | Y | Y | Y | Y | Y | Y | Y | Y | 9 |
| Alwan K, et al 2015 | Y | Y | NA | Y | Y | Y | Y | Y | Y | 9 |
| BUTA. W, 2016 | Y | NA | Y | Y | Y | Y | Y | Y | Y | 8 |
| Asemahagn, Mulusew A 2016 | Y | Y | Y | Y | Y | Y | Y | NA | NA | 7 |

**Key: Y** = Yes; **NR** = Not reported, **NA** = Not appropriate

Question codes.

1. Was the sample frame appropriate to address the target population?
2. Were study participants sampled in an appropriate way?
3. Was the sample size adequate?
4. Were the study subjects and the setting described in detail?
5. Was the data analysis conducted with sufficient coverage of the identified sample?
6. Were valid methods used for the identification of the condition?
7. Was the condition measured in a standard, reliable way for all participants?
8. Was there appropriate statistical analysis?
9. was the response rate adequate, and if not, was the low response rate managed appropriately?
